# Increased liver AGEs induce hepatic injury mediated through an OST48 pathway

**DOI:** 10.1038/s41598-017-12548-4

**Published:** 2017-09-25

**Authors:** Aowen Zhuang, Felicia YT. Yap, Clinton Bruce, Chris Leung, Manuel R. Plan, Mitchell A. Sullivan, Chandana Herath, Domenica McCarthy, Karly C. Sourris, Phillip Kantharidis, Melinda T. Coughlan, Mark A. Febbraio, Mark P. Hodson, Matthew J. Watt, Peter Angus, Benjamin L. Schulz, Josephine M. Forbes

**Affiliations:** 1 Glycation and Diabetes, Mater Research Institute – The University of Queensland, Translational Research Institute, Woolloongabba, Australia; 2 School of Medicine, University of Queensland, St Lucia, Australia; 3 Diabetic Complications Group, Baker IDI Heart and Diabetes Institute, Melbourne, Australia; 4 Department of Immunology and Medicine, Central and Eastern Clinical School, AMREP Precinct, Monash University, Clayton, Australia; 5 Institute for Physical Activity and Nutrition (IPAN), Deakin University, Burwood, Australia; 6 Department of Medicine, University of Melbourne, Austin Hospital, Heidelberg, Australia; 7 Metabolomics Australia, Australian Institute for Bioengineering and Nanotechnology, University of Queensland, St Lucia, Australia; 8 Centre for Nutrition and Food Science, Queensland Alliance for Agriculture and Food Innovation, University of Queensland, St Lucia, Australia; 9 School of Pharmacy, University of Queensland, Woolloongabba, Australia; 10 Biomedicine Discovery Program and the Department of Physiology, Monash University, Clayton, Australia; 11 School of Chemistry and Molecular Biosciences, University of Queensland, St Lucia, Australia; 12 Mater Clinical School, University of Queensland, St Lucia, Australia

## Abstract

The protein oligosaccharyltransferase-48 (OST48) is integral to protein N-glycosylation in the endoplasmic reticulum (ER) but is also postulated to act as a membrane localised clearance receptor for advanced glycation end-products (AGE). Hepatic ER stress and AGE accumulation are each implicated in liver injury. Hence the objective of this study was to increase the expression of OST48 and examine the effects on hepatic function and structure. Groups of 8 week old male mice (n = 10–12/group) over-expressing the gene for OST48, dolichyl-diphosphooligosaccharide-protein glycosyltransferase (*DDOST*+/−), were followed for 24 weeks, while randomised to diets either low or high in AGE content. By week 24 of the study, either increasing OST48 expression or consumption of high AGE diet impaired liver function and modestly increased hepatic fibrosis, but their combination significantly exacerbated liver injury in the absence of steatosis. *DDOST*+/− mice had increased both portal delivery and accumulation of hepatic AGEs leading to central adiposity, insulin secretory defects, shifted fuel usage to fatty and ketoacids, as well as hepatic glycogen accumulation causing hepatomegaly along with hepatic ER and oxidative stress. This study revealed a novel role of the OST48 and AGE axis in hepatic injury through ER stress, changes in fuel utilisation and glucose intolerance.

## Introduction

Chronic liver disease is increasing globally as a result of obesity and diabetes, with non-alcoholic fatty liver disease (NAFLD), the most common liver disorder^1^. However, NAFLD is asymptomatic and most individuals do not progress to non-alcoholic steatohepatitis (NASH). The development of NASH is postulated to be the result of a ‘second hit’ environmental factor^2^.

Glucose intolerance and dyslipidaemia which are often characteristic of NAFLD^3^, facilitate the non-enzymatic post-translational modification of proteins, forming advanced glycation end products (AGEs)^4^, which accumulate in tissues and contribute to liver fibrogenesis^4^. Excessive exposure to exogenous sources of AGEs, such as from food, can also exacerbate liver injury in the absence of glucose intolerance^5^. This is likely via simple diffusion through the epithelium if cleaved^6^, although AGE specific receptors may facilitate receptor-mediated gastro-intestinal trafficking. Once absorbed, AGEs are directed to the liver through the portal vein and excreted via the gall bladder by hepatic sinusoidal Kupffer and endothelial cells^7^, and by renal clearance^8^.

The liver, however, is not only a site for the clearance of AGEs, but also a target organ and expresses various AGE receptors including the receptor for advanced glycation end-products (RAGE)^9^, advanced glycation end product-receptor 1 (OST48 also known as AGE-R1)^10^ and galectin-3 (AGE-R3)^11^. There is accumulating evidence which suggests that in NAFLD, AGEs are a ‘second hit’ that triggers progression from steatosis to NASH^12^. Galectin-3 (AGE-R3) has also been associated with inflammation and liver fibrosis^11^ and a Phase 2a multi-centre trial is underway in individuals with portal hypertension and NASH cirrhosis (NCT02462967), using galectin-3 inhibitors. Although the studies to date in galectin-3 have focused on its immunomodulatory roles, it is feasible that AGE binding and signalling via galectin-3 is also prevented by galectin-3 inhibition contributing to the efficacy of these agents in preventing liver pathology.

The effects of AGEs are mediated by their receptors, which the effects can be broadly contrasting depending on the type of receptor. For example RAGE facilitates oxidative stress, cell growth and inflammation^13^. Specifically, in chronic liver injury, hepatic expression of RAGE is significantly increased^14^ and several studies in acute liver injury have identified that blockade of RAGE can ameliorate toxic, ischemic and cholestatic liver damage^15^. Alternatively, another AGE receptor, OST48, is thought to be responsible for the detoxification and clearance of AGEs and negative regulation of AGE pro-inflammatory signalling^16,17^, and cellular oxidative stress^18^. A decline in the expression of OST48 associated with increases in AGEs has been demonstrated both in murine models^19^ and in individuals with diabetes^17^. Although OST48 is postulated as an AGE clearance receptor, its primary role is within the endoplasmic reticulum (ER) lumen^20^ where as a subunit of the multiprotein oligosaccharyltransferase complex it facilitates enzymatic *N*-linked glycosylation of selected asparagine residues during protein translation^21^.

The present studies have demonstrated that OST48, a protein that was previously thought to mediate its effects via N-glycosylation or detoxification and clearance of AGEs, is rather a facilitator of increased AGE deposition in the liver. Specifically, a novel OST48-AGE pathway that leads to the onset of liver injury in combination with central adiposity and glucose intolerance, despite ample physical activity. These results establish a previously unappreciated physiological trafficking role of OST48 in the gastrointestinal tract and liver, which when dysregulated result in liver injury.

## Results

### Generation of a ubiquitous OST48 knock-in mouse model

OST48 knock-in mice (*ROSA26* ^*tm1*(*DDOST*)*Jfo*^ hereon termed as *DDOST*+/−) were heterozygous in their expression of human *DDOST* at the ROSA26 locus under the control of the ubiquitin promoter (Fig. S1A). There was a significant increase in hepatic OST48 gene (*DDOST*) expression (Fig. S1B), whilst endogenous gene expression (*Ddost*) was unaffected (Fig. S1C). Targeted proteomics identified that 32 week old *DDOST*+/− mice did not show a significant change in total OST48 protein in the gut using the major peptides detected (Fig. S1D). However, there is a substantial increase in OST48 protein in each major section of the gut than compared to an average of 5 differently tissue locations (Fig. S1D). Moreover, in fractionated liver tissue taken from *DDOST*+/− mice at the same time point, there was a significant increase in plasma membrane localisation of *DDOST*+/− fed on a high AGE diet (Fig. S1E). This increase in plasma membrane OST48 protein localisation and content in liver taken from *DDOST*+/− high AGE fed mice was not seen in the cytosol (data not shown). The small intestine (duodenum, jejunum and ileum) had a high relative abundance of OST48 protein when compared with other tissues (Fig. S1D).

### In the absence of steatosis, hepatic injury was evident in *DDOST*+/− mice and this was exacerbated by a high AGE diet

**Figure 1:**
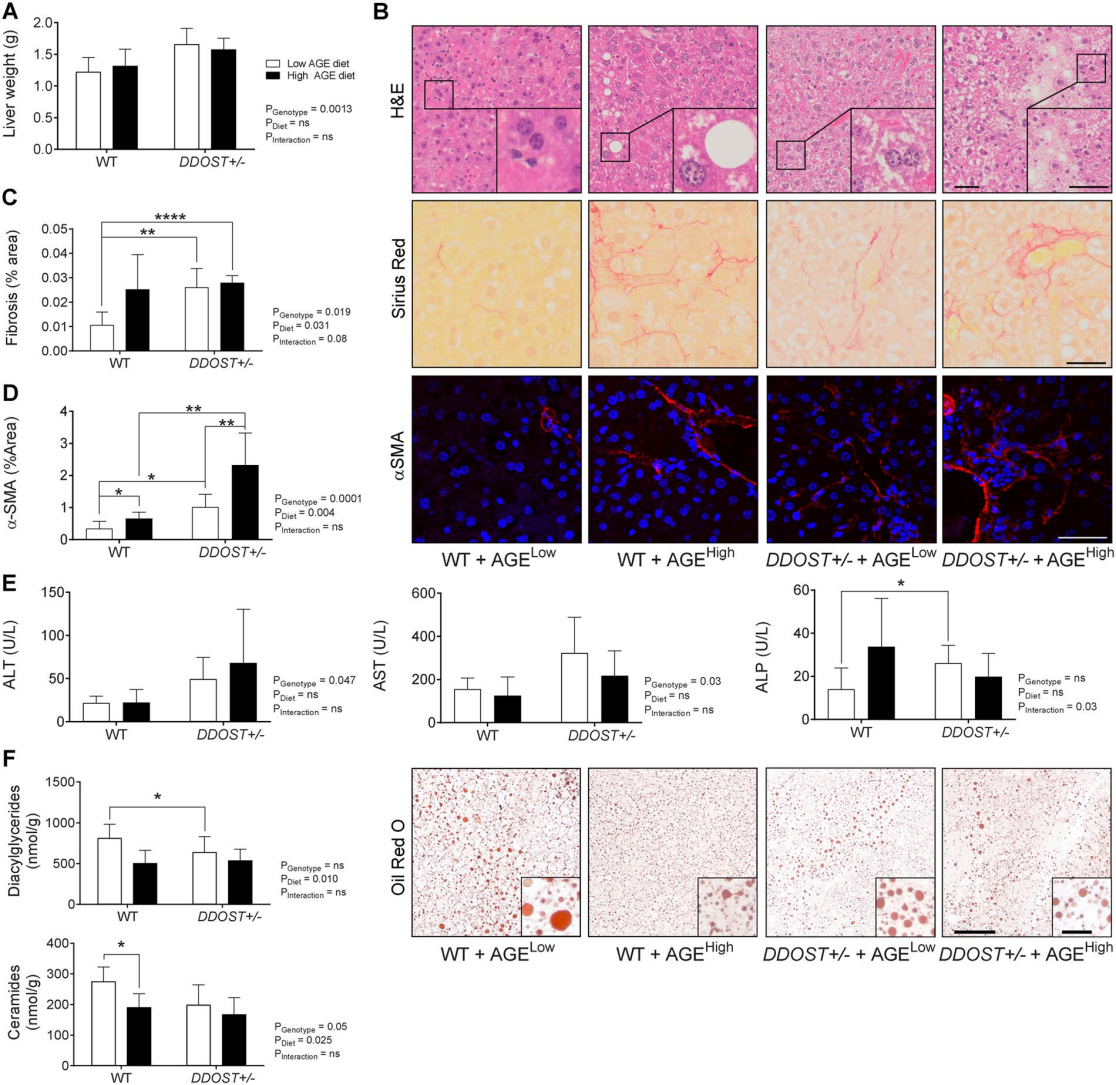
A diet high in AGE content is a promoter of portal vein fibrosis following up-regulation of *DDOST*+/− in the absence of steatosis. (**A**) Cull liver weight. (**B**) Representative H & E stained paraffin-embedded liver tissue sections. (**C**) Hepatic fibrillar collagen quantification of Sirius Red stained images (left). Representative images of fibrosis stained images of Sirius Red localised around fibrillar fibrosis extending between hepatocytes (right). (**D**) Hepatic α-SMA positive quantification of immunofluorescent stained images (left). Representative images of α-SMA positive staining extending between hepatocytes (right). (**E**) Liver function tests for ALT (left), AST (middle) and ALP (right). (**F**) Hepatic diacylglyceride (top) and ceramide (bottom) accumulation. Representative images of Oil Red O staining for lipid droplets in liver tissue (right). Data represented as means ± SD (*n* = 4–9/group). *P < 0.05, student’s t-test. Genotype effect *P* < 0.05, (diet effect) *P* < 0.05, 2-way ANOVA and multiple comparison of genotype, diet and interaction by Bonferroni’s post hoc test. Representative images scale bar = 50 µm (outside box) and 20µm (inside box).

Hepatomegaly was evident in *DDOST*+/− mice irrespective of the diet (Fig. 1A). H&E staining indicated that wild-type mice on a high AGE content diet had some hepatocellular ballooning, with modest rarefication of the cytoplasm (Fig. 1B). All *DDOST*+/− mice exhibited hepatocellular enlargement and ballooning, clusters of lobular plasma cells, increased inflammatory infiltration and an abundance of rarefied cytoplasm in hepatocytes (Fig. 1B), as well as increases in hepatic fibrosis exhibited by increased Sirius Red staining and positive α-SMA staining, respectively, (Fig. 1C and D). Although there was no substantial differences in Sirius Red staining between high AGE fed WT mice and *DDOST*+/− mice (Fig. 1C). We could however observe a gradual exacerbation of α-SMA staining in high AGE fed *DDOS*T+/− mice (Fig. 1D). Plasma concentrations of the liver enzymes ALT and AST were also increased in *DDOST*+/− mice (Fig. 1E), and plasma ALP concentrations were the highest in *DDOST*+/− mice fed a high AGE diet, suggesting the presence of hepatocellular damage.

Hepatocellular ballooning is often indicative of lipid droplet accumulation in the liver. Despite the presence of vascular fibrosis, there were no differences among groups in hepatic Oil Red O staining for lipid droplets (Fig. 1F; right). Further, there was a significant reduction in both hepatic DAGs (Fig. 1F; top; *P* = 0.0101) and ceramides (Fig. 1F; bottom; *P* = 0.0246) associated with high AGE dietary intake. There was also a modest decrease (−30.9% low AGE diet and −15.9% high AGE diet, *P* = 0.0516, 2-way ANOVA) in hepatic ceramide content when comparing the *DDOST*+/− genotype with the WT mice however, this did not reach statistical significance.

### *DDOST*+/− mice exhibited increased absorption of AGEs in the liver

**Figure 2:**
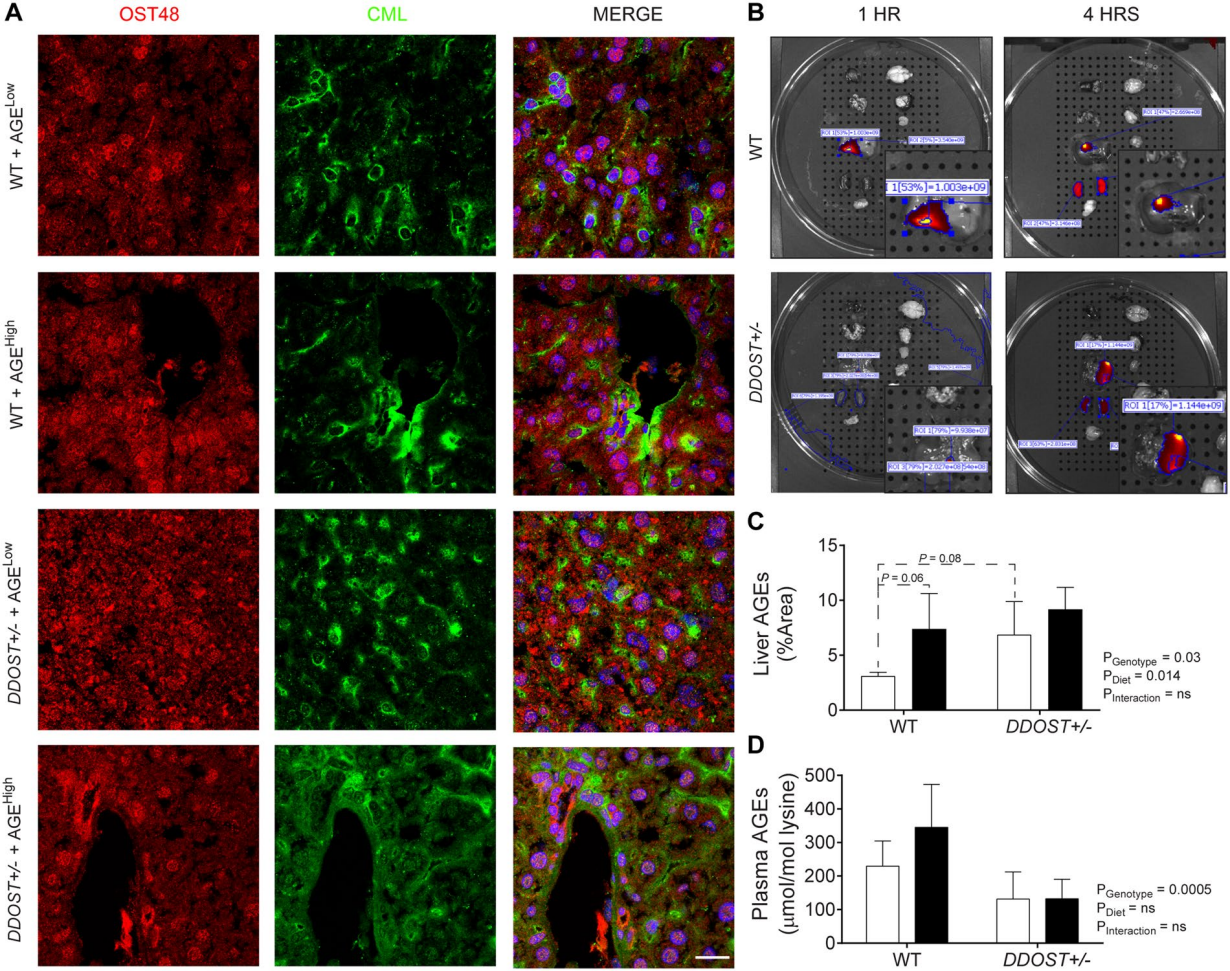
*DDOST*+/− mice have increased hepatic uptake of AGEs. Wild-type and *DDOST*+/− mice were fed either a high AGE (baked AIN-93G diet) or a low AGE (unbaked AIN-93G) diet for 24 weeks. (**A**) Immunofluorescence for OST48 (red) and the AGE, CML (green) in OCT liver sections. Representative images; scale bar = 20 µm (**B**) Near-infrared imaging of AGE uptake in the liver following oral gavage using IVIS/MRI. (**C**) AGE concentrations in hepatic tissue by immunofluorescence quantification. (**D**) Plasma AGE concentrations by ELISA. Data represented as means ± SD (*n* = 4–9/group). Genotype effect *P* < 0.05, 2-way ANOVA and multiple comparison of genotype, diet and interaction by Bonferroni’s post hoc test.

The two-hit model of a high AGE diet and *DDOST*+/− mice exhibited increased deposition of both CML and OST48 in hepatic tissue sections (Fig. 2A). Oral administration of near-infrared labelled AGEs using IVIS/MR imaging, suggested prolonged exposure of AGEs in the liver of *DDOST*+/− mice (Fig. 2B). We further identified that hepatic AGE deposition was greater in mice fed a high AGE diet and in *DDOST*+/− mice irrespective of dietary alterations (Fig. 2C). Although WT mice fed a high AGE diet had similar deposition of AGEs in the liver, it was identified that circulating AGE concentrations were reduced in all *DDOST*+/− mice as compared with high AGE fed littermate WT mice (Fig. 2D) indicating an increase in trafficking and exposure of AGEs from the circulation in the liver.

### Despite the absence of steatosis *DDOST*+/− mice had increased adiposity in addition to increased physical activity

**Figure 3:**
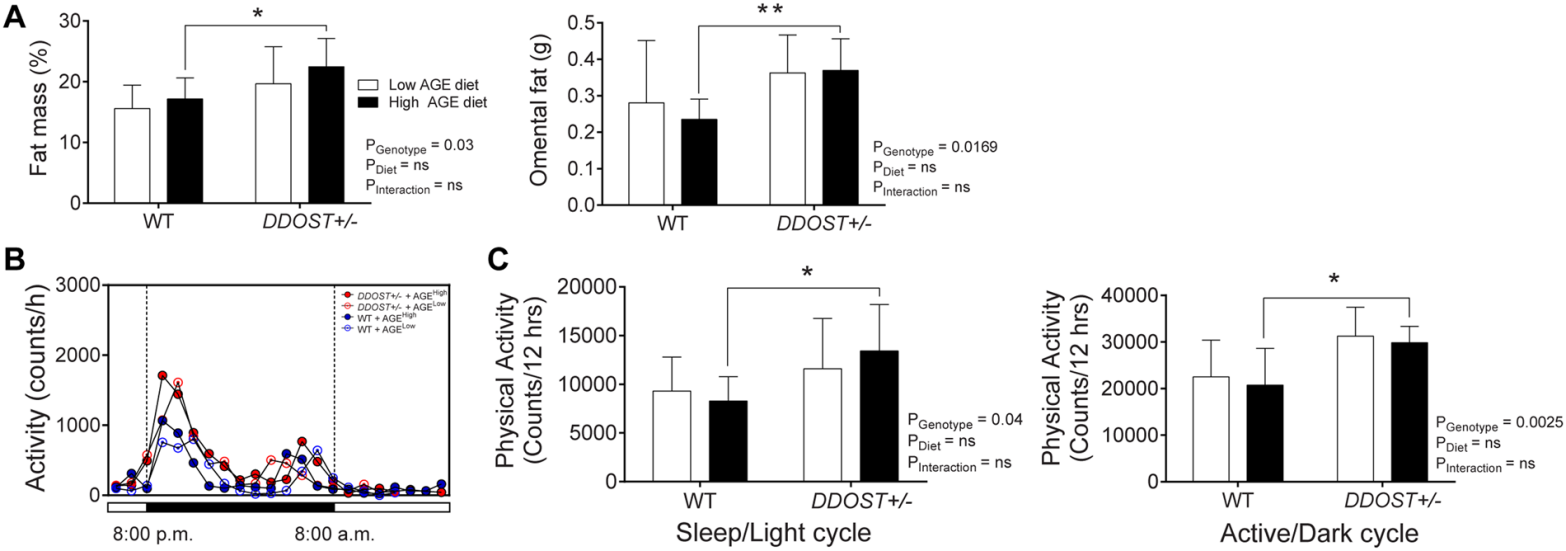
*DDOST*+/− mice have increased central adiposity despite greater physical activity. (**A**) Fat percentage measured by EchoMRI (left) and weight of omental fat deposits (right). (**B**,**C**) CLAMS apparatus measured 24 hour physical activity and caloric intake at 24 weeks post-dietary intervention in WT () and *DDOST*+/− () mice fed on either a high AGE diet (closed circles) or a low AGE diet (open circles). (**B**) Total locomotor activity in the horizontal plane measured by infrared beam breaks. (**C**) The line graphs show average hourly heat generation over the 24-hour period. The bar graphs on the right show the average physical activity over the entire 12-hour light/sleep (left) and dark/active (right) period. Data represented as means ± SD (*n* = 4–9/group). *P < 0.05, student’s t-test, α (genotype effect) P < 0.05, β (diet effect) P < 0.05, as per figure above. 2-way ANOVA and multiple comparison of genotype, diet and interaction by Bonferroni’s post hoc test.

There were no differences in body weight or lean body mass at either the beginning (Fig. S2A and B) or the end of the study (Fig. S2D and E) among mouse groups. Body fat mass was increased in *DDOST*+/− mice as compared to their respective WT group (Fig. 3A; left), and this was more pronounced with high AGE feeding at the study end. Indeed, adiposity, measured as the intra-abdominal fat pads (mesenteric and omental fat pads), was increased in *DDOST*+/− mice as compared to WT littermates (Fig. 3A; right). At the beginning of the study, there were no significant differences in adiposity between WT and *DDOST*+/− mice (Fig. S2C). Increased adiposity in *DDOST*+/− mice was not a consequence of reduced physical activity (Fig. 3B; left), nor of increased food intake (Fig. S2F). In fact, *DDOST*+/− mice exhibited increased locomotor activity during both dark/awake and light/sleep phases (Fig. 3C; right), irrespective of diet.

### Hepatic injury associates with ER stress, oxidative stress and activated inflammasome

**Figure 4:**
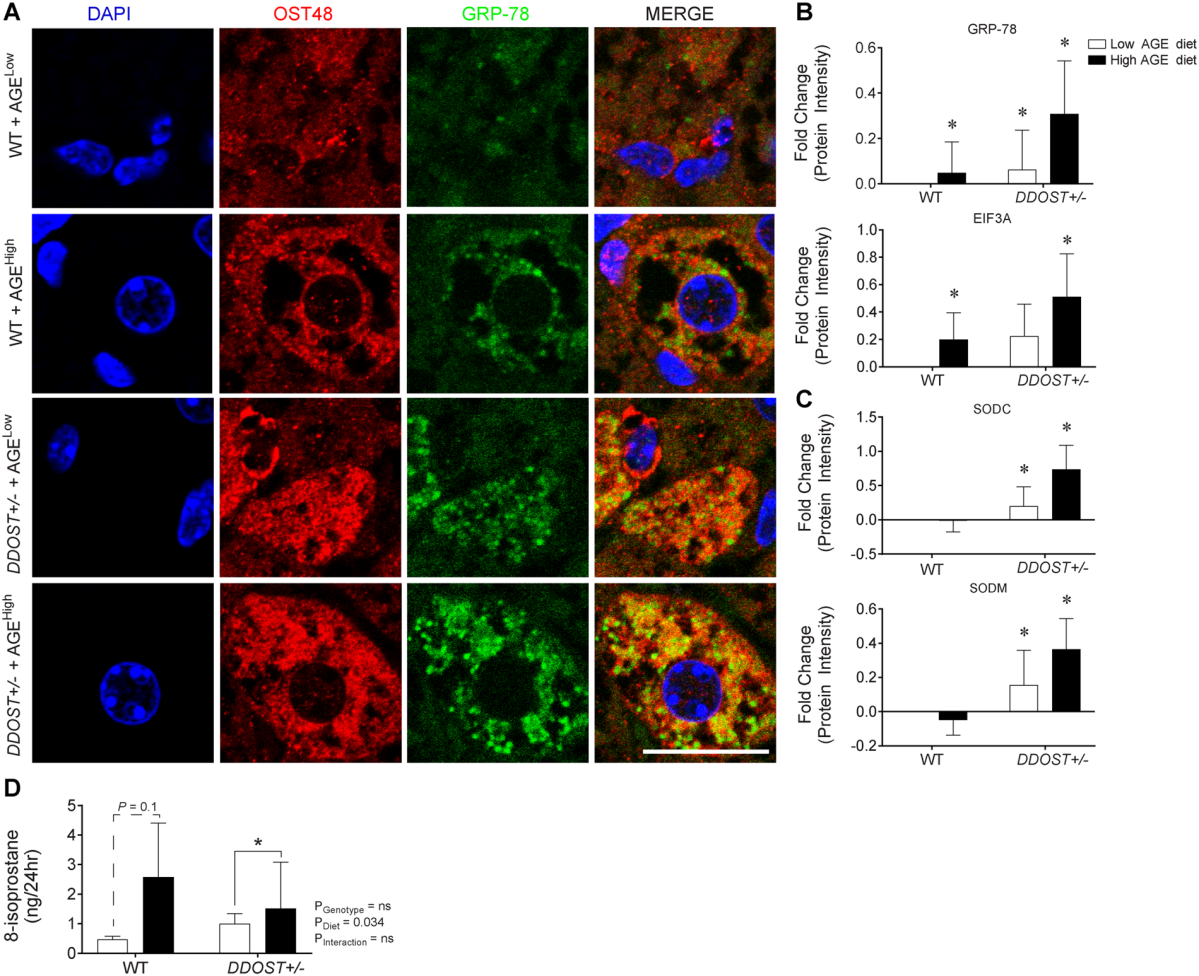
A high AGE diet promotes liver fibrosis and correlates with increased ER stress markers following increases in *DDOST*+/− mice. (**A**) Immunofluorescence for OST48 (red), the ER stress marker GRP-78 (green) on paraffin liver sections (hepatocytes). (**B**) SWATH-MS protein intensities of ER stress pathway related proteins. GRP78 (top) and EIF3A (bottom). (**C**) SWATH-MS protein intensities of oxidative stress pathway related proteins. SODC (top) and SODM (bottom). (**D**) ELISA identifying content of urinary 8-isoprostane. Data represented as means ± SD (*n* = 4–9/group). *P < 0.05, MSstatsV3.5.1 determined significant log fold changes in the protein intensities between the selected experimental group and the WT low AGE diet group. Representative images scale bar = 20 µm.

Confocal microscopy, showed co-localisation of GRP-78 with OST48 (Fig. 4A). Unbiased proteomic profiling of hepatic tissue using SWATH-MS, identified increases in key proteins involved in ER stress, specifically GRP78 (Fig. 4B; top) and EIF3A (Fig. 4B; bottom) in our two-hit model of OST48 over-expression and high AGE feeding. The anti-oxidant enzymes SOD1/SODC (Fig. 4C; top) and SOD2/SODM (Fig. 4C; bottom) were increased in *DDOST*+/− mice irrespective of the diet consumed. *DDOST*+/− mice fed a low AGE diet also had increases in the redox responsive GPX1 (Fig. S3A; left) and aconitase/ACON (Fig. S3A; right) when compared to low AGE fed WT mice. Furthermore, we also observed a significant (*P* = 0.034) effect of a high AGE diet on increased 8-isoprostane levels in the urine (Fig. 4D). *DDOST*+/− mice fed a high AGE diet also had significant increases in the inflammatory proteins CD47 (Fig. S3B; left) and HA1D (Fig. S3B; right).

### Defects in N-glycosylation do not explain the hepatic injury seen in *DDOST*+/− mice fed on a high AGE diet

Prolonged ER stress culminating in liver injury is often associated with impaired ER function leading to changes in glycosylation occupancy on proteins. We therefore investigated whether *DDOST*+/− mice altered hepatic protein synthesis N-glycosylation. Fifty-six glycosylation sites were identified on mouse plasma proteins (Fig. S4A) with six indicating partial modification by N-glycosylation (Fig. S4B), but there were no differences among groups in glycosylation occupancy on plasma proteins (Fig. S4A), nor in plasma proteins specifically glycosylated and secreted by the liver (Fig. S4C).

### Hepatic injury was associated with changes in fatty acid metabolism

**Figure 5:**
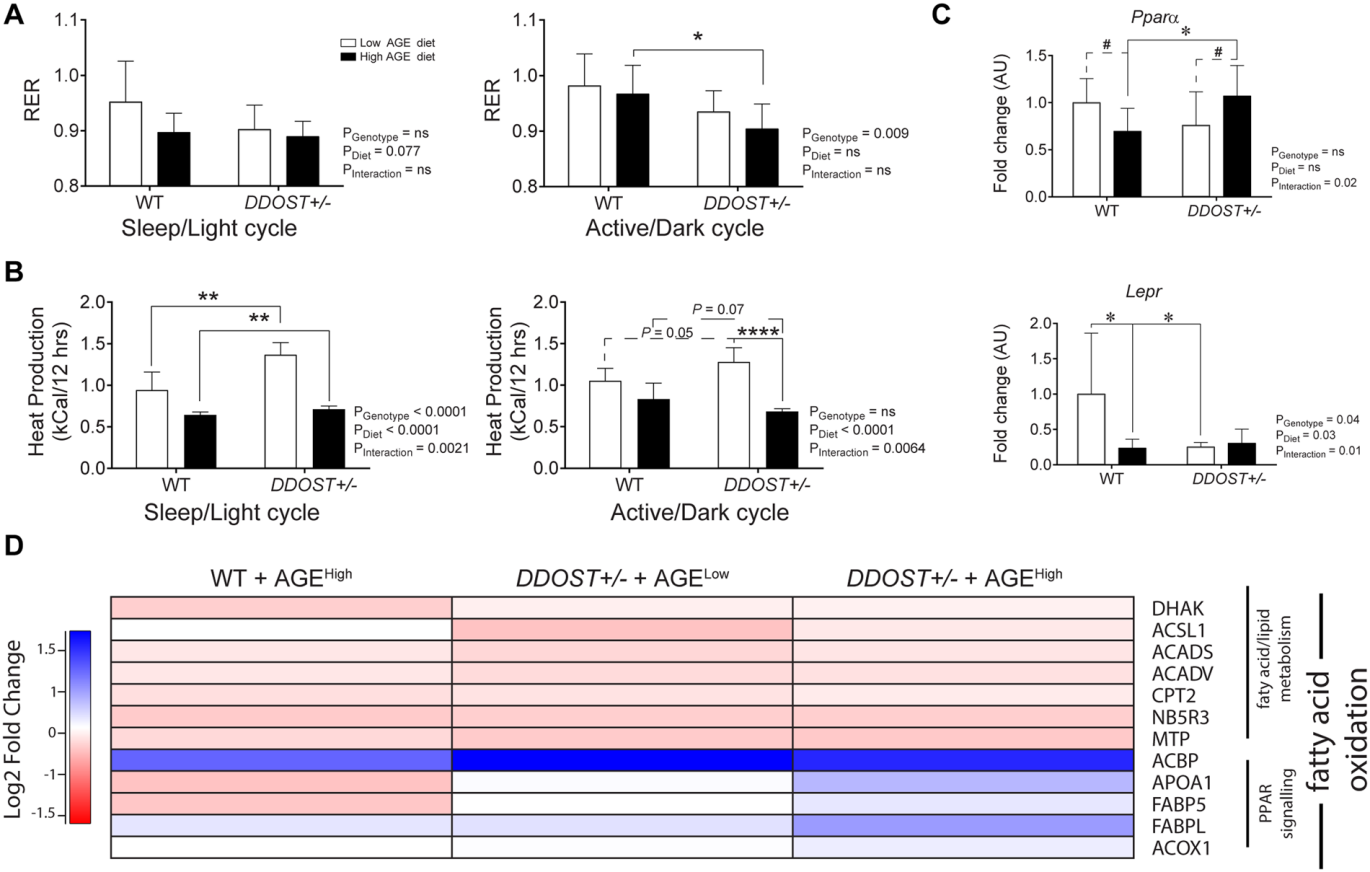
*DDOST*+/− mice have a shift towards increased fatty acid utilization. (**A**) CLAMS apparatus measured respiratory exchange rate at 24 weeks post-diet modification. Average difference in respiratory quotient corrected to lean body mass measured by indirect calorimetry during the entire 12-hour light/sleep (left) and dark/active (right) period. (**B**) Average difference in heat production measured by indirect calorimetry during the entire 12-hour light/sleep (left) and dark/active (right) period. (**C**) Real-time PCR of liver tissue targeting genes of interest *Pparα* (top) and *Lepr* (bottom) (*n* = 4–8 per group in triplicate). (**D**) Heat map representation of SWATH-MS proteomics data for enzymatic pathways involved in fatty acid oxidation. Significant proteins are represented as the Log_2_ fold change where red indicates a decreased and blue indicates an increase in protein concentrations. Data represented as means ± SD (*n* = 4–9/group). For proteomics, MSstatsV3.5.1 determined significant (*P* < 0.05) log fold changes in the protein intensities between the selected experimental group and the WT low AGE diet group. *P < 0.05, student’s t-test. Genotype effect *P* < 0.05, (diet effect) *P* < 0.05, 2-way ANOVA and multiple comparison of genotype, diet and interaction by Bonferroni’s post hoc test.

Using continuous indirect calorimetry, we determined that *DDOST*+/− mice had decreased respiratory exchange ratios during both the sleep/light (Fig. 5A; left) and active/dark (Fig. 5A; right) phases. This downward shift in RER suggested a proportional shift towards the use of free fatty acids and/or ketones for ATP generation as compared to wild-type littermates consuming either a low/high AGE diet. In support of this, an RER of 0.9, seen in *DDOST*+/− mice on a high AGE diet during their active cycle, demonstrates a ~65:35 of carbohydrate:fat/ketone oxidation as compared with WT mice fed a low AGE diet, (RER of 0.98; ~93:7 carbohydrate:fat/ketone oxidation)^22^. During the light cycle, *DDOST*+/− mice fed a high AGE diet had an RER of 0.88, demonstrating a large shift towards fatty acid and ketone oxidation (~59:41 carbohydrate:fat/ketone oxidation) as compared with low AGE fed WT mice (RER of 0.95; ~83:17 carbohydrate:fat/ketone oxidation). Moreover, we observed significant decreases in heat generation during both the sleep/light (Fig. 5B; left) and active/dark phases (Fig. 5B; right) specifically associated with a high AGE diet (*P* < 0.0001) in both WT and *DDOST*+/− mice.

The gene expression of key enzymes involved in fatty acid utilisation by the liver were altered in *DDOST*+/− mice fed a high AGE diet (Fig. 5C). Specifically, there was a significant increase in *Pparα* gene expression in *DDOST*+/− mice fed a high AGE diet (Fig. 5C; top) as compared with other groups. The gene expression of *Lepr* (Fig. 5C; bottom) was decreased by both the high AGE diet and in all *DDOST*+/− mice. *DDOST*+/− high AGE fed mice, also showed a decrease in the gene expression of both *Acadvl* (Fig. S5; left) and *Acadm* (Fig. S5; right), encoding enzymes involved in the oxidation of medium and long chain fatty acids.

Using SWATH-MS proteomics, significant increases in proteins associated with fatty acid oxidation, peroxisome proliferator-activated receptor (PPAR) protein signaling and fatty acid transport/export were demonstrated in *DDOST*+/− mice fed a high AGE diet when compared with WT low AGE fed mice (Fig. 5D). Specifically, the fatty acid oxidation enzymes ACOX1 and ACBP were increased, as well as the transport proteins FABP5 and FABPL, and the HDL core protein APOA1. The other proteins associated with lipid metabolism examined were not significantly altered.

### *DDOST*+/− mice exhibit impaired glucose tolerance at a young age

Young (8 week old) *DDOST*+/− mice had increases in fasted blood glucose concentrations (Table 1 and Fig. S6A), lower fasting insulin concentrations and decreases in first phase insulin secretion (Table 1 and Fig. S6B) during ipGTT as compared with WT littermates. There were no differences in fed blood glucose concentrations (Table 1), or in insulin tolerance tests (Fig. S6C) among young mice.

**Table 1:** Biochemical measurements in mice post-diet modifications (32 weeks of age).

Post-diet modification							
	WT		*DDOST*+/−		Two-Way ANOVA		
	AGE^Low^	AGE^High^	AGE^Low^	AGE^High^	G	D	G∙D
Fed Glucose (mmol/L)	15.70 ± 1.80	13.77 ± 1.53	15.66 ± 1.38	13.95 ± 1.18	0.96	0.23	0.94
Fasting Glucose (mmol/L)	10.65 ± 0.46	9.417 ± 1.13	9.913 ± 0.76	10.03 ± 0.47	0.94	0.49	0.96
Fasting Insulin (ng/ml)	1.255 ± 0.41	1.837 ± 0.53	0.9438 ± 0.27	0.7999 ± 0.14	**0.05**	0.51	0.28
Fasting Glucagon (pg/ml)	1366 ± 187.8	2087 ± 287.6	1552 ± 80.13	1677 ± 279.8	0.61	0.09	0.93

Mean and standard deviation for biochemical measurements of each genotype during pre- and post-diet modification. Post-diet modification significance levels were determined by two-way ANOVA, testing the effect of genotype and diet. Differences between variables identified by Bonferroni’s post hoc test. Bold *P* values indicates significant effect of at least < 0.05. G: Genotype; D: Diet; G∙D: Interaction.

### Adult *DDOST*+/− mice exhibited glucose intolerance which is augmented by high AGE dietary intake

**Figure 6:**
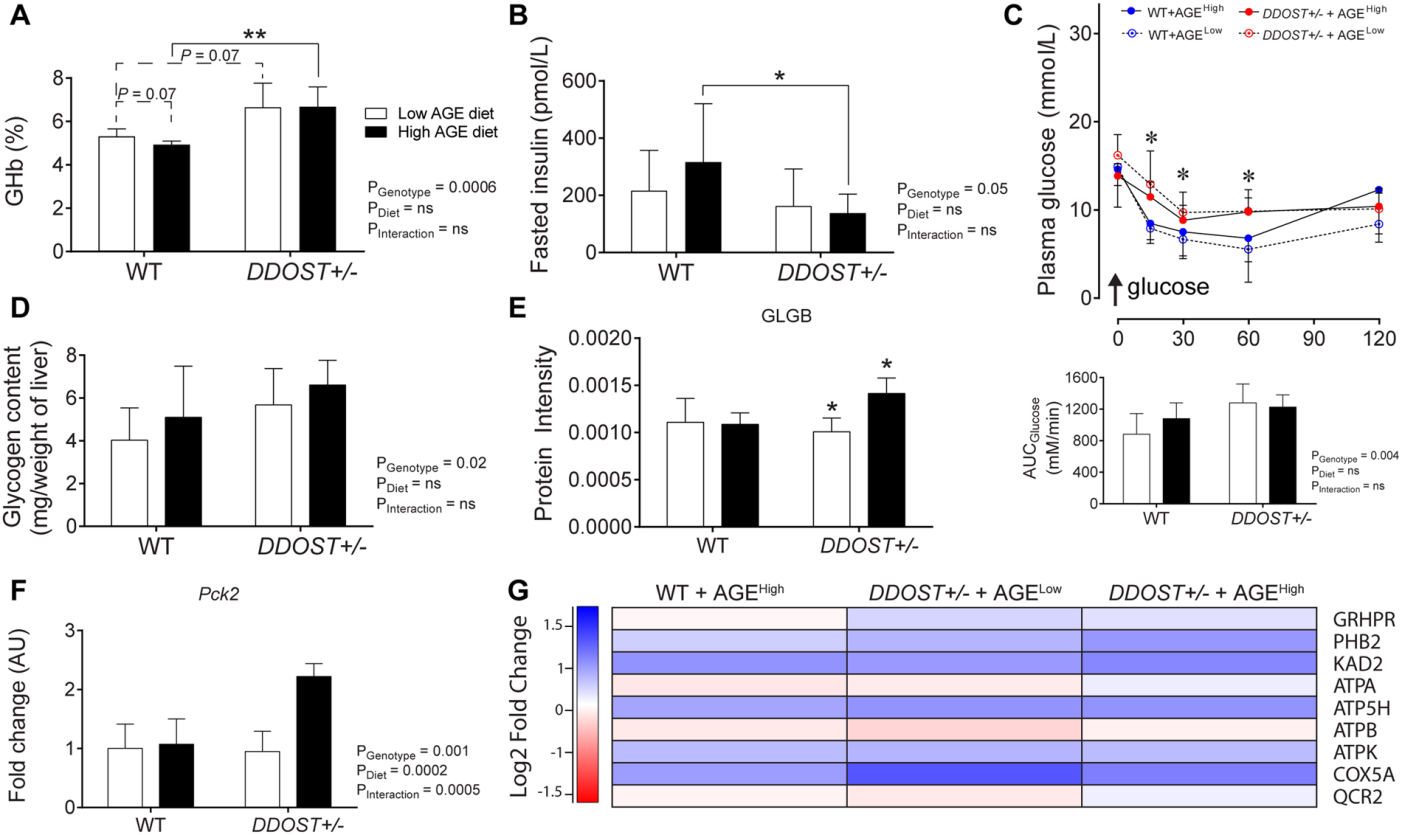
Hepatic fibrosis associated with glucose intolerance, increased glucose synthesis and storage in high AGE fed *DDOST*+/− mice. (**A**) Glycated haemoglobin. (**B**) Fasting plasma insulin concentrations. (**C**) Plasma glucose curve over 120 mins following a 1IU/kg insulin bolus (ipITT) and area-under-the-curve (AUC) analysis. (**D**) Hepatic glycogen measured in liver tissue. (**E**) SWATH-MS protein intensities for the glycogen storage pathway protein (GLGB). (**F**) qPCR of *Pck2*, the enzyme involved in gluconeogenesis. (**G**) Heat map representation of SWATH-MS proteomics data for enzymatic pathways involved in GNG. Significant proteins are represented as the Log_2_ fold change where red indicates a decreased and blue indicates an increase in protein concentrations. Data represented as means ± SD (*n* = 4–9/group). For proteomics, MSstatsV3.5.1 determined significant (*P* < 0.05) log fold changes in the protein intensities between the selected experimental group the WT low AGE diet group. *P < 0.05, student’s t-test. Genotype effect *P* < 0.05, (diet effect) *P* < 0.05, 2-way ANOVA and multiple comparison of genotype, diet and interaction by Bonferroni’s post hoc test.

By the end of the study, all *DDOST*+/− mice had elevated glycated haemoglobin (Fig. 6A) and lower fasted plasma insulin concentrations (Fig. 6B), without differences in fasted or fed plasma glucose concentrations as compared with WT mice (Table 1). However, high AGE fed *DDOST*+/− mice also had increased 2 hour plasma glucose concentrations following a 2 g/kg glucose bolus (ipGTT), as compared with other groups (Fig. S7A and B). While on a high AGE diet, WT mice were less glucose tolerant as compared with low AGE fed mice (Fig. S7C).

In response to an insulin bolus (ipITT), *DDOST*+/− mice fed a high AGE diet had higher plasma glucose concentrations than other groups (Fig. 6C). Fasting plasma glucagon concentrations were not increased (Table 1), but hepatic glycogen storage was increased by 30–40% in *DDOST*+/− mice (Fig. 6D). When compared with the WT low AGE fed group, all mice also had lower protein intensities of 1,4-alpha-glucan-branching enzyme (GLGB), a facilitator of glycogenolysis (Fig. 6E), consistent with greater levels of liver glycogen described above.

The gene expression of the gluconeogenic enzyme, *pyruvate carboxylase kinase* (*Pck*2; Fig. 6F) was increased by high AGE dietary feeding. Pathway analysis of proteins involved in GNG showed that hepatic proteins involved in oxidative phosphorylation were elevated in all mouse groups when compared with WT low AGE fed mice (Fig. 6G).

### *DDOST*+/− mice on a high AGE diet exhibit increased amino acid metabolism

**Figure 7:**
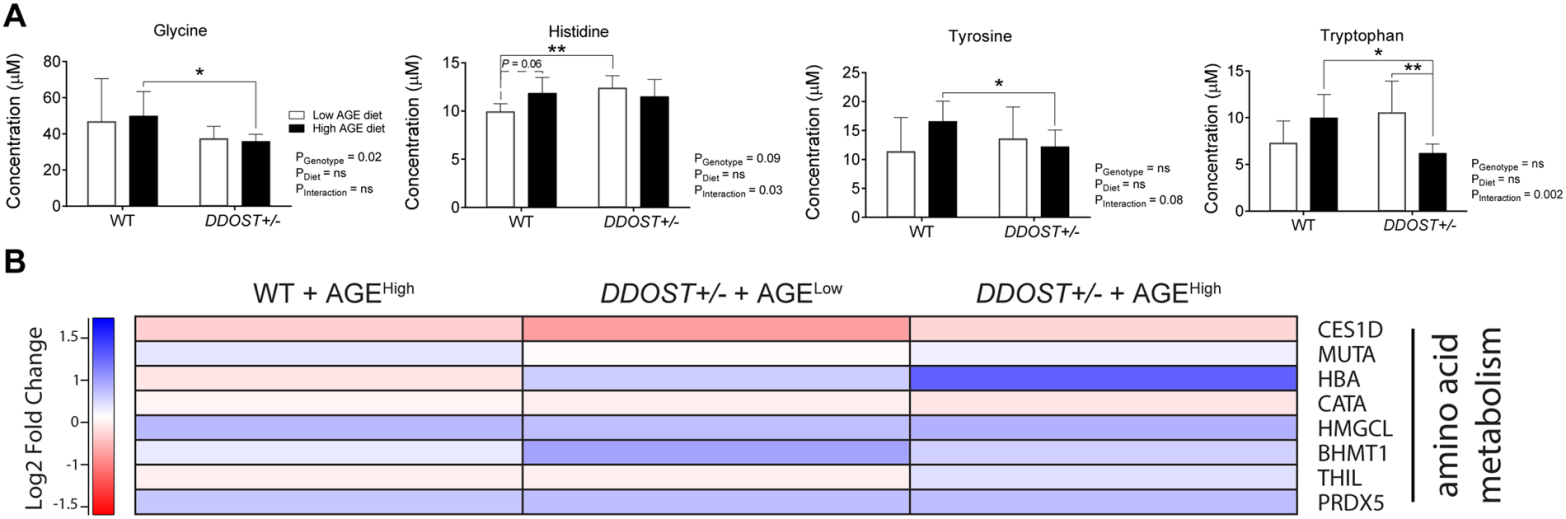
*DDOST*+/− mice exhibit increased ketogenesis. (**A**) Concentrations of serum amino acids. (**B**) Heat map representation of SWATH-MS proteomics data for enzymatic pathways involved in ketogenesis. Significant proteins are represented as the Log_2_ fold change where red indicates a decreased and blue indicates an increase in protein concentrations. Data represented as means ± SD (*n* = 4–9/group). For proteomics, MSstatsV3.5.1 determined significant (*P* < 0.05) log fold changes in the protein intensities between the selected experimental group the WT low AGE diet group.

Increased gluconeogenesis and hepatic glucose release to potentially sustain an associated increased physical activity can be further substantiated by the evident increased circulating concentrations of the amino acids alanine (Fig. 7A) and serine (Fig. 7B) and the loss of glucogenic glycine in *DDOST*+/− mice (Fig. 7A). Plasma concentrations of the essential amino acids lysine and histidine (Fig. 7C) were also increased in *DDOST*+/− mice. Plasma concentrations of both tyrosine and tryptophan were also increased in the high AGE fed *DDOST*+/− mice when compared with other mouse groups (Fig. 7D). Pathway analyses showed an increase in protein associated with amino acid metabolism in high AGE fed *DDOST*+/− mice (Fig. 7E). There were no other changes observed in circulating concentration of other amino acids (Fig. S8).

## Discussion

Our discovery of the accumulation of AGEs in the liver through an OST48 mediated pathway of uptake and clearance is novel. Despite the unconventional pathway of liver injury (normally the presence of steatosis leading to the onset of fibrosis) in our *DDOST*+/− mice, this allowed us to identify other metabolic factors that may contribute an important role in the development of liver injury^23^. A recent study in children with NAFLD demonstrated that the presence of early portal inflammation on biopsy was independently associated with severity of liver fibrosis and metabolic risk factors for type 2 diabetes, in particular waist circumference^24^. Indeed in that study, central adiposity was the only non-invasive variable associated with biopsy proven portal inflammation. These findings are consistent with the present study, where central adiposity was seen in concert with early liver injury and portal fibrosis in *DDOST*+/− mice fed a high AGE diet. Whether *DDOST*+/− mice bypass the development of steatosis and proceed directly into fibrogenic pathways would require further investigation into a high fat diet model. Despite this caveat what was particularly interesting was that central adiposity and liver injury persisted in *DDOST*+/− mice fed a high AGE diet, despite them partaking in greater levels of physical activity. This interesting finding reiterates the complexity of the relationship between metabolic factors and liver injury, given that exercise is a feasible intervention to improve liver fibrosis in patients with NAFLD^25^ and relates to adiposity in NAFLD^26^.

The liver is a node which controls systemic glucose and lipid fluxes in the body, balancing these against its own energy requirements^27^. These processes are influenced heavily by pancreatic islet function via the endocrine hormones insulin and glucagon^28^ as well as other hormones. In the present study, abnormalities in insulin secretion and elevations in glycated haemoglobin were seen in the context of hepatic injury and increased fuel oxidation, particularly of fatty acids in high AGE fed *DDOST*+/− mice. This is in agreement with previous evidence that a shift in hepatic fuel utilisation towards fatty acids results in liver damage^29^. The increased utilisation of fatty acids for β-oxidation, and likely increased fatty acid export/transport proteins, may also explain the lack of hepatic fat accumulation seen in *DDOST*+/− mice fed a high AGE diet. Surprisingly, this led to an increase in central adiposity, despite increased physical activity and the absence of changes in caloric intake and lean body mass. There also appeared to be an excess supply of circulating glucose generated from hepatic gluconeogenesis which may be required by the skeletal muscle to facilitate the increases in physical activity seen in *DDOST*+/− mice, rather than for thermogenesis. This is also supported by the decreases in heat generation seen in AGE-R1 mice fed a high AGE diet. A futile cycle of energy generation in adipocytes has been linked to reduced body fat storage^30^, however it has not been previously linked to the development of liver fibrosis.

In summary, this group of studies revealed a previously unappreciated role of the OST48-AGE axis within the body. Indeed, the data suggest that the “trafficking role” of OST48 in normal hepatic physiology is not solely involved in protein N-glycosylation or elimination (detoxification/clearance) of AGEs but could also alter gastrointestinal uptake through the small intestine of AGEs leading to changes in glucose homeostasis, hepatic function and fuel utilisation. The physiological significance of this should form the basis of future studies. In the context of a high AGE diet, increases in OST48 expression exacerbated liver injury leading to fibrosis, likely via known pathways of injury including oxidative and ER stress. Further studies into the role of hepatic and gastrointestinal OST48 mediated AGE uptake could lead to a novel approach to minimise liver injury.

## Materials and Methods

### Animal Model

C57BL/6J mice (The Jackson Laboratory, United States) were genetically modified via the *Cre*-*loxP* recombination system (Ozgene, WA, Australia) and ubiquitous genetic knock-in of the human gene encoding OST48 (*DDOST*) at the ROSA26 locus with removal of the delivery neomycin cassette, as described in *Supporting Experimental Procedure 1* and *Supporting* Fig. 1. *DDOST* ^*flox*/*flox*^ mice are referred to as the wild-type (WT) background and *DDOST* ^*flox*•*Cre*/*flox*^ are labelled as the genetic OST48 heterozygous knock-in mouse model (*DDOST*+/−). Eight week old male *DDOST*+/− mice and littermate controls (WT) were randomised to be fed either AIN-93G (low AGE diet; Specialty Feeds, Perth, Australia)^31^ or baked AIN-93G (1 hour at 100 °C; high AGE diet), which contained a 5-fold higher content of the AGEs, N(ε)-(carboxymethyl)lysine (CML), N(ε)-(carboxyethyl)lysine and methylglyoxal^32^ for 24 weeks. Heat labile vitamin contents (vitamin A and thiamine) were not decreased by the heating protocol^32^. Mice were allowed access to food and water *ad libitum* and were maintained on a 12 hour light:dark cycle at 22 °C. All mouse experiments were performed following approval from the AMREP Animal Ethics Committee and as per guidelines from the National Health and Medical Research Council of Australia.

### Plasma AGE measurement

CML concentrations in plasma were measured by an in-house indirect ELISA as previously described^33^.

### Human and mouse OST48 ELISAs

Mouse and human OST48 concentrations were determined in hepatic cortical membrane and cytosolic fractions by commercial sandwich ELISAs (Cloud-Clone Corp., Houston, United States), according to the manufacturer’s specifications.

### Liquid Chromatography-Mass Spectrometry (LC-MS/MS)

As previously described^34^, proteins were extracted from whole liver tissue samples using guanidine denaturing buffer (6 M guanidinium, 10 mM DTT and 50 mM Tris-HCl). Reduced cysteines were alkylated with acrylamide, and quenched with excess DTT. Proteins were precipitated in 4 volumes of 1:1 methanol:acetone and digested with trypsin. Peptides were desalted and analysed by Information Dependent Acquisition LC-MS/MS as described^35^ using a Prominence nanoLC system (Shimadzu, NSW, Australia) and Triple TOF 5600 mass spectrometer with a Nanospray III interface (SCIEX). SWATH-MS analysis was performed^36^ and analysed with MSstats as previously described. Differentially abundant proteins were analysed using DAVID^37^.

### Histology and Immunofluorescence

Haemotoxylin and Eosin, Masson’s Trichrome and Sirius Red (Sigma-Aldrich, United States) stains were completed on 10% Buffered formalin fixed paraffin sections. Collagen content of the liver was quantified histologically using computerised quantification of picrosirius red staining, as described previously^15^. Oil-Red-O (Sigma-Aldrich, USA) staining was performed on frozen OCT embedded cryosections as previously described^38^. All sections were visualized on a slide scanner (Virtual Slide System VS120, Olympus, Tokyo, Japan) and viewed in the supplied program (OlyVIA Build 10555, Olympus, Tokyo, Japan). Briefly, for immunofluorescence staining, 4% PFA fixed frozen liver sections were dual stained with both anti-CML (1:125 dilution; ab30917; Abcam, United Kingdom) and OST48 (1:100 dilution; sc25558; Santa Cruz biotechnologies, United States). Dual staining on paraffin buffered formalin fixed sections was with GRP78 (1:50 dilution; sc1050; Santa Cruz biotechnologies, United States) and OST-48 (1:100 dilution; sc74407; Santa Cruz biotechnologies, United States).

### Serum and urine biochemistry and analysis of hepatic lipids

The plasma concentrations of alanine transaminase (ALT), aspartate transaminase (AST) and alkaline phosphatase (ALP) were measured by an auto-analyzer (Beckman Instruments, USA). Hepatic diacylglycerols (DAGs) and ceramides were extracted and quantified by thin layer chromatography and a liquid scintillation analyzer (LS6500; Beckmann Coulter Inc., CA, USA) as previously described^39^. Lipid peroxidation was examined by urinary 8-isoprostane in a competitive ELISA (Oxford Biomedical Research, United States), according to the manufacturer’s specifications.

### Glucose and insulin tolerance tests

Intraperitoneal glucose tolerance tests (ipGTT) were performed following a 2 g/kg D-glucose bolus as previously described^40^. Insulin was determined by rat/mouse insulin ELISA (RnD systems, MN, United States). Intraperitoneal insulin tolerance test (ipITT), to determine glucose output was performed using a 1 IU/kg bolus of Humulog fast-acting insulin (Eli Lilly, IN, United States). Area under the curve was calculated using the trapezoidal rule (GraphPad Software, CA, United States).

### Indirect calorimetry and assessment of body composition

Whole body composition was measured at 32-weeks of age in conscious, but physically restrained mice using an EchoMRI™ 3-in-1 body composition analyzer (EchoMRI, TX, USA). A cohort of mice were allocated for repeated measures of energy expenditure by indirect calorimetry (VO_2_, VCO_2_) normalised to lean body mass, locomotor/physical activity and rate of energy expenditure (heat generation) using a Comprehensive Lab Animal Monitoring System (CLAMS; Columbus Instruments, OH, United States). Data was collected over a 24-hour cycle period, post-acclimatisation to the cages for a 12-hour period.

### Liver glycogen content

As previously described^41^, total liver glycogen content was determined using a glucose oxidase/peroxidase assay procedure and absorbance was analysed on a UV-1700 PharmaSpec UV-vis spectrophotometer (Shimadzu, NSW, Australia).

### Quantitative real-time PCR

Messenger RNA was purified and was used (1 µg) to synthesize cDNA using SuperScript first-strand synthesis system (Invitrogen). Quantitative real-time PCR was performed using pre-designed TaqMan Gene Expression Assays® for *Acadm*, *Acadvl*, *Acox1*, *ADRB2*, *Ccl2*, *Col1a1*, *Col3a1*, *Ddost*, *DDOST*, *G6pc*, *Gcgr*, *Got1*, *Gyk*, *Kcnma1*, *Lepr*, *Pck1*, *Pck2*, *Ppara*, *Slc27a4* and *Slc37a4* (*Supporting Experimental Procedure 2*; Life Technologies, Mulgrave, VIC, Australia) in ViiA™ 7 real-time PCR system (Applied Biosystem, Darmstadt, Germany). Gene expression levels were calculated after normalisation to an endogenous multiplexed control (18S) using the ΔΔCT method as previously described^32^ and expressed as relative fold change compared to the wild-type mice fed a high AGE content diet. The results are represented as mean ± %CV.

### Amino acid measurements

Approximately 40 µl of serum was mixed with 160 µl of an extraction solution (3:1 ratio of analytical grade methanol to ddH_2_O), centrifuged at 16,000 × *g* for 10 minutes and the supernatant removed for processing. Amino acids were measured by HPLC as described in Chacko *et al*.^42^.

### Statistical analysis

Results are expressed as mean ± SD (standard deviation), and analyzed by 2-way ANOVA followed by post hoc testing for multiple comparisons using the Bonferroni method unless otherwise specified. α (genotype effect) *P* < 0.05, β (diet effect) *P* < 0.05, δ (interaction effect) *P* < 0.05 were reported. For comparison between groups as required, a two-tailed unpaired Student’s t-test was used. For SWATH-MS, MSstatsV3.5.1 was used to detect differentially abundant proteins estimating the log-fold changes between compared conditions of the chosen experimental group and with the WT mice fed a low AGE diet group. For all calculations a *P* < 0.05 was considered as statistically significant.

## Electronic supplementary material

Supplementary Information
