# Supplementary material for: Increased liver AGEs induce hepatic injury mediated through an OST48 pathway
Source: Sci Rep. 2017 Sep 25;7:12292. doi: 10.1038/s41598-017-12548-4 (PMC5612946; doi:10.1038/s41598-017-12548-4)
Supplement: Supplementary file 1 — Supplementary Information [file 41598_2017_12548_MOESM1_ESM.doc]

**Title:** Increased gut absorption of AGEs mediated through an N-glycosylation pathway induces hepatic injury.

**Authors:** Aowen Zhuang BBiomSc(Hons)^1,2^, Felicia YT Yap PhD^3,4^, Clinton Bruce PhD^5^, Chris Leung MBBS^6^, Manuel R Plan PhD^7^, Mitchell A Sullivan PhD^8^, Chandana Herath PhD^6^, Domenica McCarthy BSc^1^, Karly C. Sourris PhD^3,4^, Phillip Kantharidis PhD^3^, Melinda T Coughlan PhD^3,4^, Mark A Febbraio PhD^3^, Mark P Hodson PhD^7,9^, Matthew J Watt PhD^10^, Peter Angus FRACP^6^, Benjamin L Schulz PhD^11^, *Josephine M Forbes PhD^1,6,12^

^*^ Refers to corresponding author

^1^Glycation and Diabetes, Mater Research Institute – The University of Queensland, Translational Research Institute, Woolloongabba, Australia

^2^School of Medicine, University of Queensland, St Lucia, Australia

^3^ Diabetic Complications Group, Baker IDI Heart and Diabetes Institute, Melbourne, Australia

^4^Department of Immunology and Medicine, Central and Eastern Clinical School, AMREP Precinct, Monash University, Australia

^5^Institute for Physical Activity and Nutrition (IPAN), Deakin University, Burwood, Australia

^6^Department of Medicine, University of Melbourne, Austin Hospital, Heidelberg, Australia

^7^Metabolomics Australia, Australian Institute for Bioengineering and Nanotechnology, University of Queensland, St Lucia, Australia

^8^Centre for Nutrition and Food Science, Queensland Alliance for Agriculture and Food Innovation, University of Queensland, St Lucia, Australia

^9^School of Pharmacy, University of Queensland, Woolloongabba, Australia

^10^Biomedicine Discovery Program and the Department of Physiology, Monash University, Clayton, Australia

^11^School of Chemistry and Molecular Biosciences, University of Queensland, St Lucia, Australia

^12^Mater Clinical School, University of Queensland, St Lucia, Australia

**Contact Information:** Professor Josephine Forbes, Program Leader, Chronic Disease Biology and Care, Mater Research Institute – UQ, Translational Research Institute, 37 Kent Street, Woolloongabba, QLD, 4102, Australia. Ph: +61734437642 Email: [Josephine.Forbes@mater.uq.edu.au](mailto:Josephine.Forbes@mater.uq.edu.au)

**Supplementary Materials and Methods**

*Genotyping of genetic knock-in mutation for DDOST+/- mice.* All genotyping was confirmed with genomic Southern blot hybridization using the ScaI and the 5’ probe. Mice were then re-screened with the same filter with a generic Cre probe to determine whether they contain the ubiquitous promoter (**Supporting Fig. 1**).

Pre-designed TaqMan Gene Expression Assays® IDs

| **Gene name** | **Life Technologies Assay ID** |
| --- | --- |
| *Acadm* | Mm01323360_g1 |
| *Acadvl* | Mm00444293_m1 |
| *Acox1* | Mm01246834_m1 |
| *ADRB2* | Hs00240532_s1 |
| *Col1a1* | Mm00801666_g1 |
| *Col3a1* | Mm01254476_m1 |
| *Ccl2* | Mm00441242_m1 |
| *Ddost* | Mm00492100_m1 |
| *DDOST* | Hs00193263_m1 |
| *G6pc* | Mm00839363_m1 |
| *Gcgr* | Mm00433546_m1 |
| *Got1* | Mm01195792_g1 |
| *Gyk* | Mm00433896_m1 |
| *Kcnma1* | Mm01268569_m1 |
| *Lepr* | Mm00440181_m1 |
| *Pck1* | Mm01247058_m1 |
| *Pck2* | Mm00551411_m1 |
| *Ppara* | Mm00440939_m1 |
| *Slc27a4* | Mm01327405_m1 |
| *Slc37a4* | Mm00484574_m1 |

**Supplementary Figure 1**

**
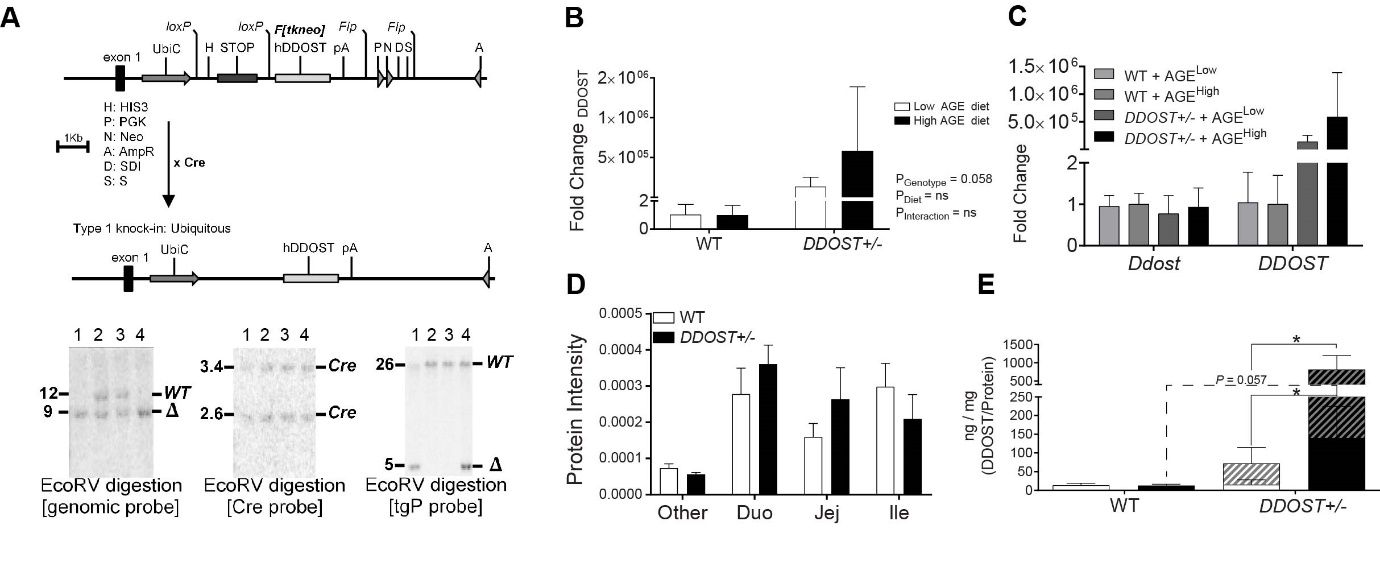
**

*Supporting Figure 1 | Generation of a ubiquitous DDOST heterozygous knock-in mutant.* (**A**) Genomic clone of DDOST bearing exon 1 used for the construction of the floxed targeting vector as indicated (**top**). Heterozygous mice were crossed with mice expressing Cre and subsequent progeny contained a ubiquitous over-expression allele. Genomic Southern blotting confirmed the predicted DDOST allelic structures in the ubiquitous knock-in strain (**bottom**). Left and middle panels: ES cell clones bearing indicated mutant DDOST allele in comparison to the parental wild-type ES cells; Right panels: adult mouse genotypes with either DDOST germline modifications or wild-type, successfully crossed with the Ubi-Cre (UbiC) strain. (**B**) Hepatic gene expression of *DDOST*, encoding for human DDOST by real-time-PCR. (**C**) Hepatic gene expression of *Ddost* and *DDOST*, encoding for mouse AGE-R1 and human AGE-R1 respectively by real-time-PCR. (**D**) Targeted SWATH proteomics for OST48 protein content in gut tissue and an average of 5 different tissues (left kidney, liver, skeletal muscle, left ventricle and epididymal fat). (**E**) Endogenous mouse DDOST and human DDOST (**striped**) protein content measured in hepatic plasma membrane by ELISA

**Supporting Figure 2**

**
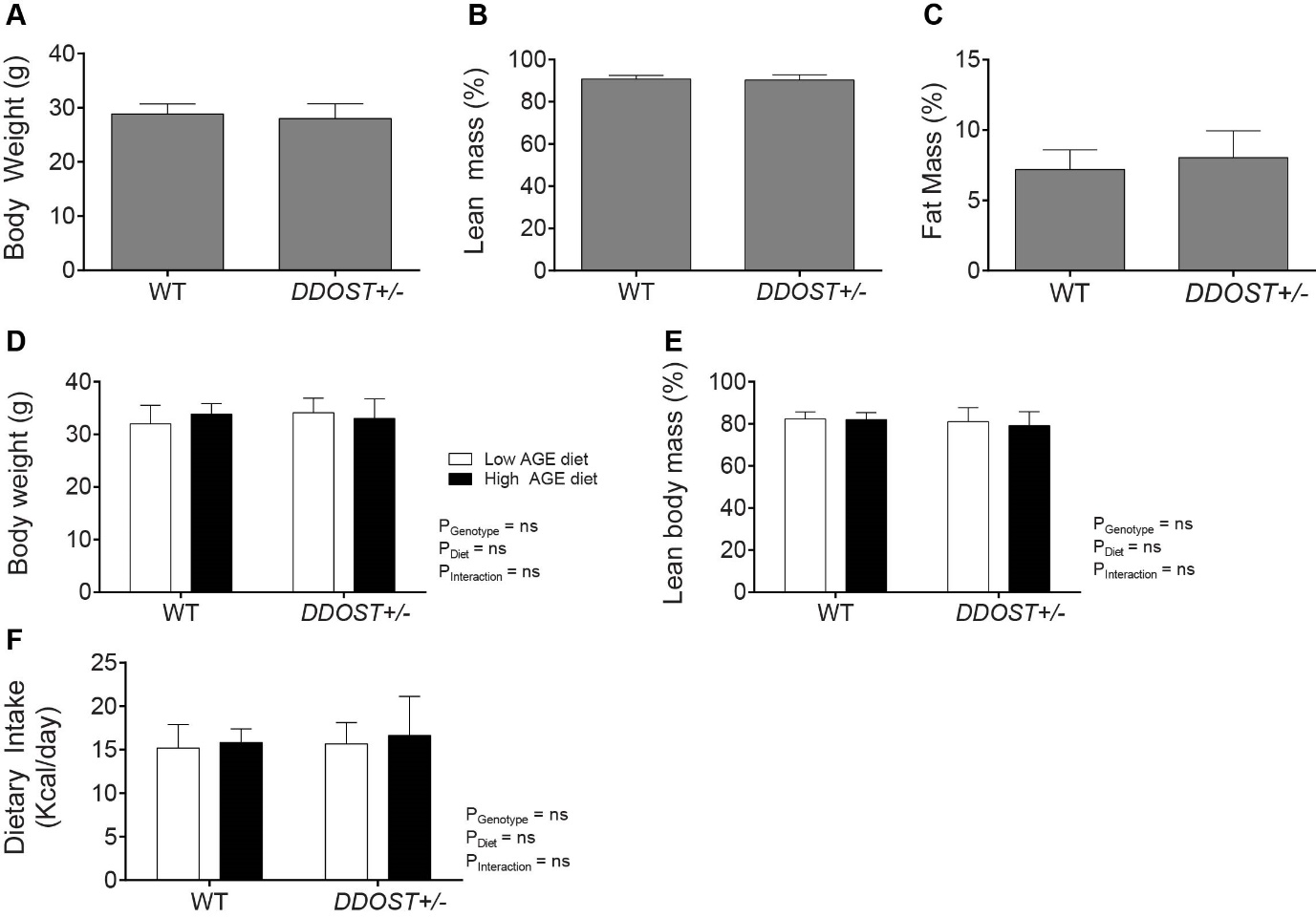
**

*Supporting Figure Legend 2 | DDOST+/- mice do not have differences in body weight or body composition pre-diet modification.* (**A**) Body weight. (**B**) Lean body mass percentage measured by EchoMRI. (**C**) Fat mass percentage measured by EchoMRI. Data represented as means ± SD (*n* = 4-9/group).

**Supporting Figure 3**

**
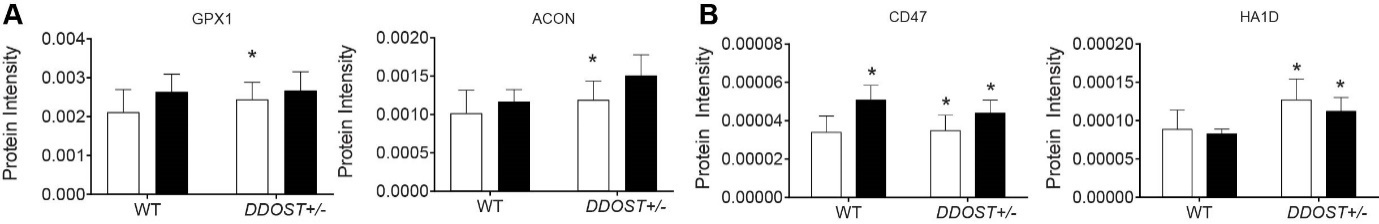
**

*Supporting Figure Legend 3 | SWATH-MS protein intensities of oxidative stress pathway related proteins and inflammatory pathway related proteins.* (**A**) GPX1 (**left**) and ACON (**right**). (**B**) CD47 (**left**) and HA1D (**right**). Data represented as means ± SD (n = 4-9/group). *P < 0.05, MSstatsV3.5.1 determined significant log fold changes in the protein intensities between the selected experimental group and the WT low AGE diet group.

**Supporting Figure 4**


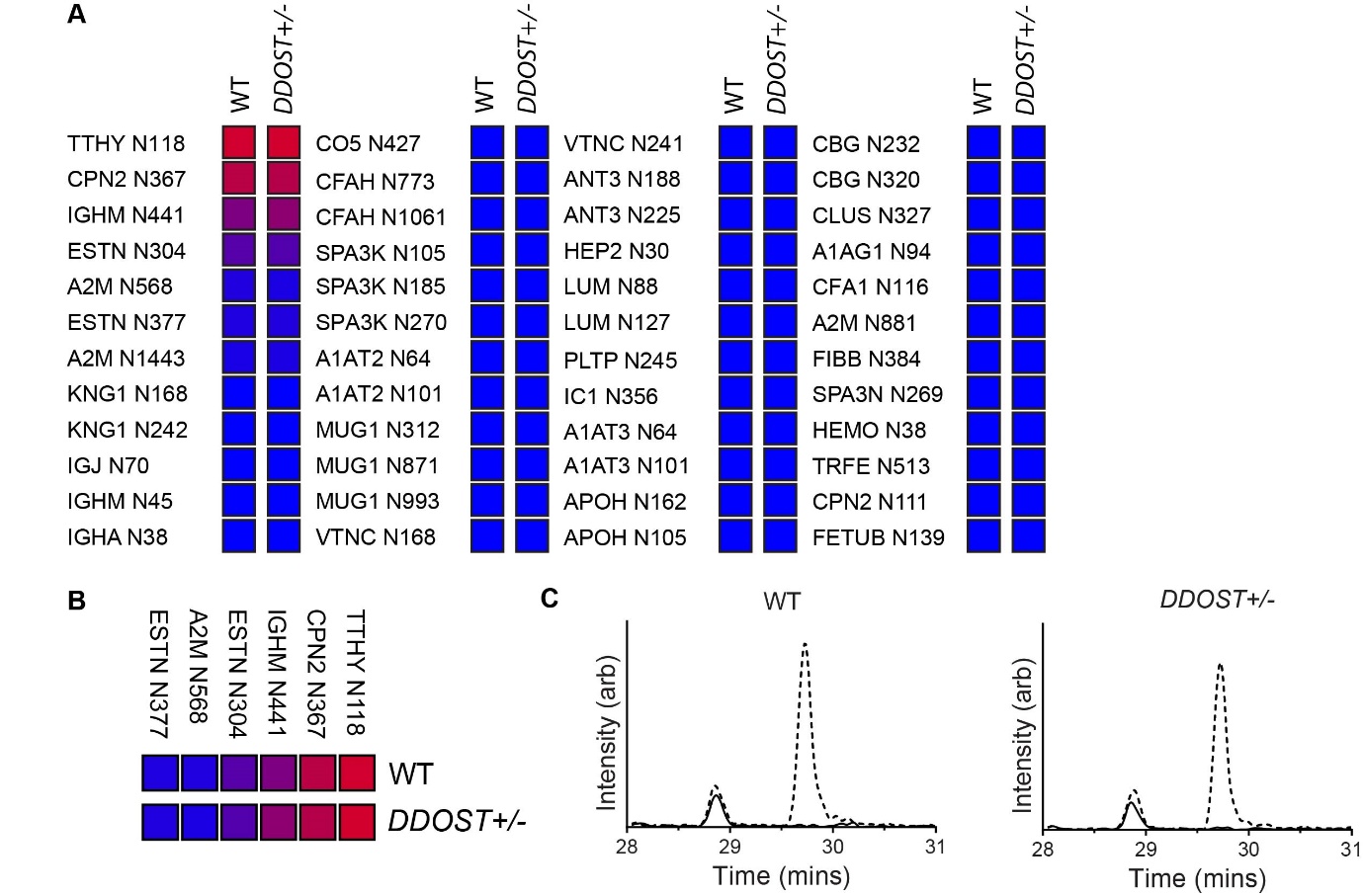


*Supporting Figure Legend 4 | Glycosylation occupancy in serum proteins.* ((**A**) Peptide-centric glycosylation occupancy at asparagine residues in mouse serum proteins measured after trypsin digest. Glycosylation occupancy was measured at different glycosylation sites (e.g. THHY N188, Asn118 in TTHY) in wild-type and *AGE-R1* mice. Color is mapped to the extent of glycosylation occupancy from 100% (blue) to 0% (red). Data is mean (*n* = 3/group of 3 replicates). (**B**) Heat map representing several proteins secreted from the liver and their degree of glycosylation occupancy. (**C**) Extracted ion chromatograms for the peptide containing ESTN N377 in liver extracts; corresponding to de-glycosylated (dashed) and non-glycosylated (solid) forms of tryptic peptide (*m/z* of 826.4297 and *m/z* of 826.1017, respectively), peak corresponds to the monoisotopic peak from each peptide.

**Supporting Figure 5**

**
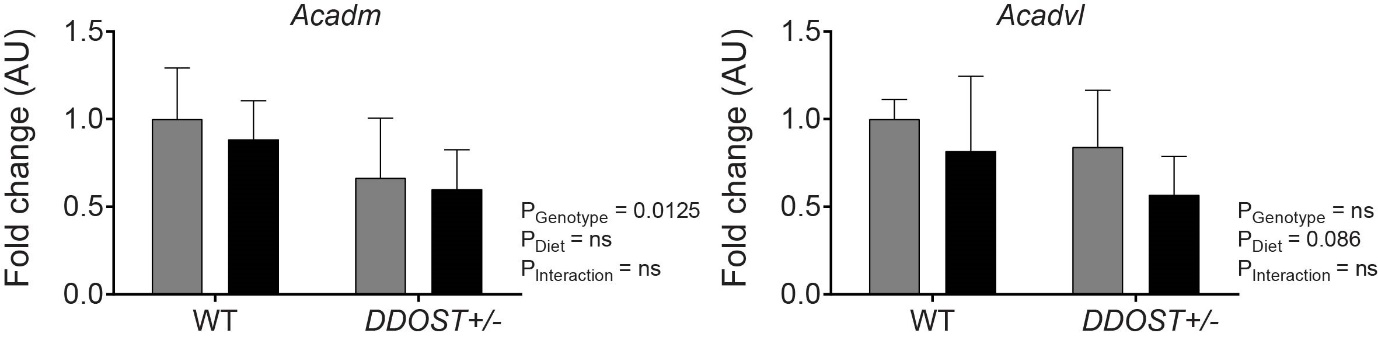
**

*Supporting Figure Legend 5* | *Real-time PCR of liver tissue targeting genes of interest*. *Acadm* (**left**) and *Acadvl* (**right**). Data represented as means ± SD (*n* = 4-8/group in triplicate).

**Supporting Figure 6**

**
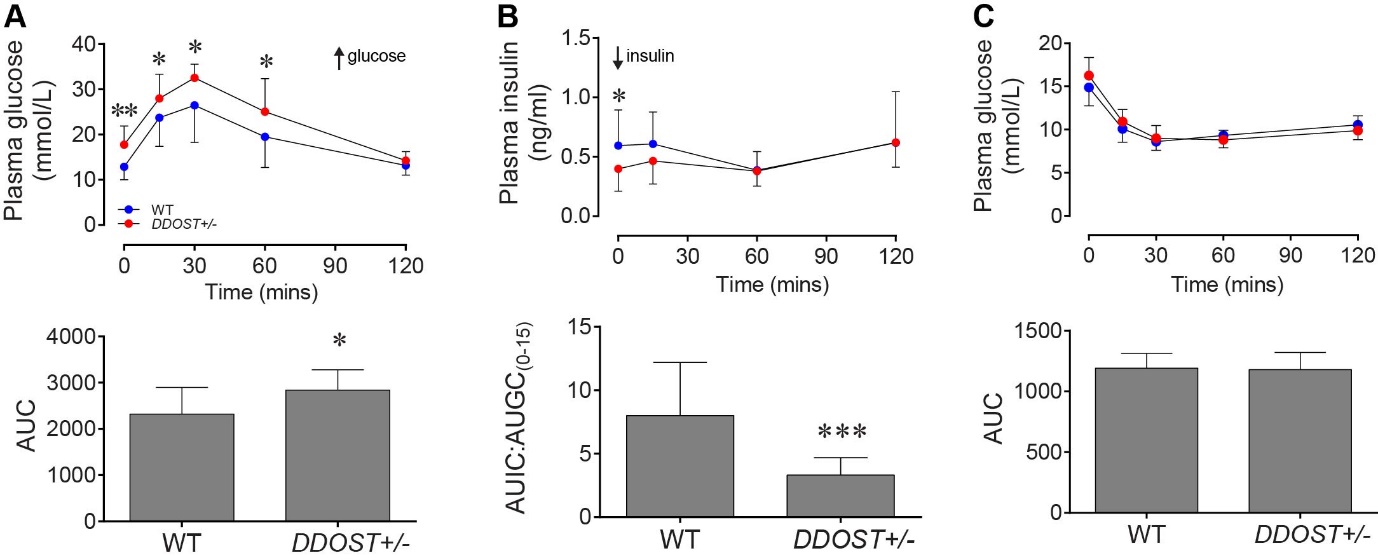
**

*Supporting Figure 6 | Young DDOST+/- mice have impaired glucose tolerance.* Young (8 week old) WT (**●**) and *AGE-R1* (**●**) mice serum glucose and insulin levels in mice fasted for 6 hours following intraperitoneal insulin or glucose injection. (**A**) ipGTT measuring glucose homeostasis (**top**) with subsequent area-under-the-curve (AUC) analysis (**bottom**). (**B**) ipITT assessing glucose effectiveness (**top**) and ratio of AUIC:AUGC of early-phase insulin response (**bottom**). (**C**) ipGTT measuring insulin effectiveness (**top**) with subsequent area-under-the-curve (AUC) analysis (**bottom**). Data represented as means ± SD (*n* = 4-9/group). **P* < 0.05, student’s t-test.

**Supporting Figure 7**


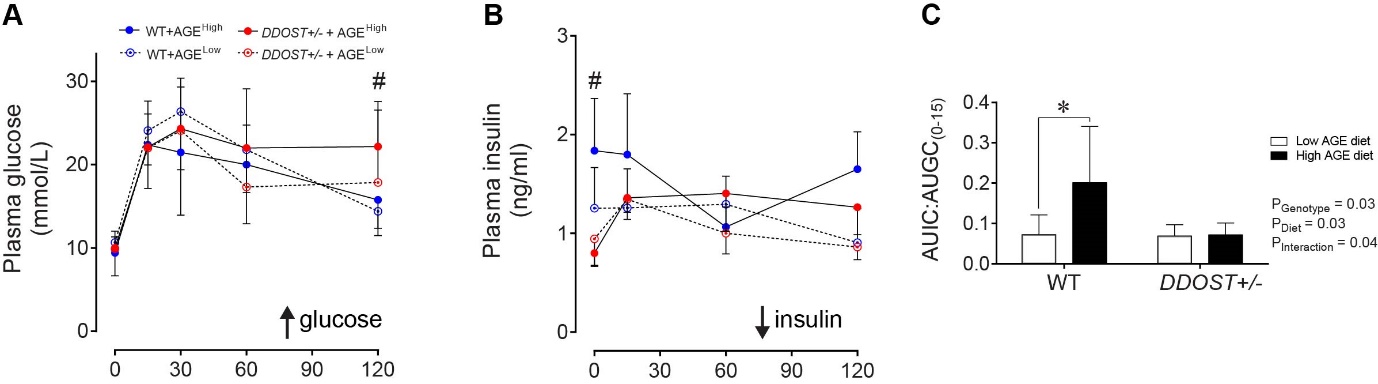


*Supporting Figure 7 | DDOST+/- mice exhibit impaired first phase insulin secretion.* (**A-B**) Intraperitoneal glucose and insulin tolerance testing in wild-type (**●**) and *DDOST+/-* (**●**) mice fasted for 6 hours after 24 weeks of high or low AGE diet feeding. (**A**) ipITT (**left**) and ipGTT (**right**) measuring glucose homeostasis and (**B**) ipGTT measuring insulin effectiveness with subsequent area-under-the-curve (AUC) analysis of serum glucose (AUGC) and insulin (AUIC). (**C**) Fasted insulin concentrations in plasma serum (**top**). Ratio of AUIC:AUGC to determine insulin effectiveness during the first phase insulin secretion response (**bottom**). Data represented as means ± SD (*n* = 4-9/group). ^#^*P* < 0.1, **P* < 0.05, ***P* < 0.01, α (genotype effect) *P* < 0.05, β (diet effect) *P* < 0.05, δ (interaction effect) *P* < 0.05, 2-way ANOVA and multiple comparison of genotype, diet and interaction by Bonferroni’s post hoc test.

**Supporting Figure 8**

**
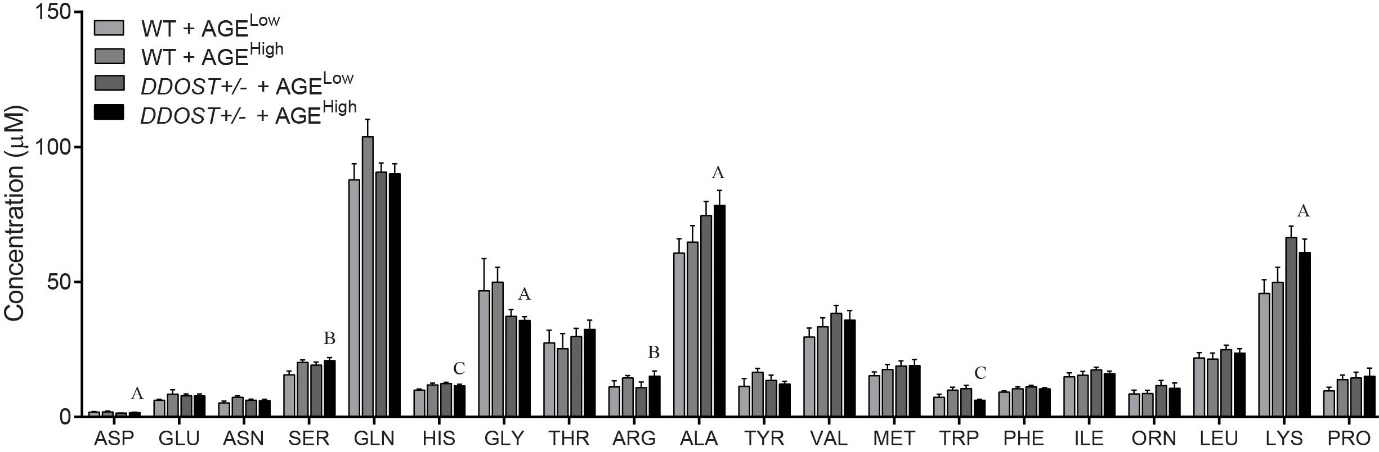
**

*Supporting Figure 8 | Serum concentrations of amino acids.* Amino acid metabolite concentration measured in plasma serum. A (genotype effect) *P* < 0.05, B (diet effect) *P* < 0.05, C (interaction effect) *P* < 0.05, 2-way ANOVA and multiple comparison of genotype, diet and interaction by Bonferroni’s post hoc test.
